# Biosimilars and reference biological medicines in the treatment of rheumatoid arthritis: a multicenter cross-sectional study in Catalonia, Spain

**DOI:** 10.1007/s10067-025-07568-9

**Published:** 2025-07-24

**Authors:** Joan Mas Marin, Marina Molina-Olano, Nuria Rudi Sola, Núria Miserachs-Aranda, Paula Montoliu Alcón, Jan T. De Pourcq, Carles Quiñones Ribas, Laura Borràs Trias, Eva Fernández-Cañabate, Juan González-Valdivieso, Carlos Figueiredo-Escribá, René Delgado-Hernández, Antonio J. Braza, Cecilia F. Lastra, Eduardo L. Mariño, Pilar Modamio

**Affiliations:** 1 Clinical Pharmacy and Pharmaceutical Care Unit, Department of Pharmacy and Pharmaceutical Technology, and Physical Chemistry, Faculty of Pharmacy and Food Sciences, University of Barcelona, Av. Joan XXIII, 27-31, 08028 Barcelona, Spain; 2 Pharmacy Service, Hospital General de Granollers, Av. Francesc Ribas s/n, 08402 Granollers, Barcelona, Spain; 3 Pharmacy Service, Fundació Hospital de l’Esperit Sant, Av. Mossèn Pons i Rabadà s/n, 08923 Santa Coloma de Gramenet, Barcelona, Spain; 4 Pharmacy Service, Hospital Universitari Sagrat Cor, C/ Viladomat, 288, 08029 Barcelona, Spain; 5 Pharmacy Service, Hospital de la Santa Creu i Sant Pau, C/ Sant Antoni Mª Claret 167, 08025 Barcelona, Spain; 6 Pharmacy Service, Hospital Universitari Germans Trias i Pujol, Carretera de Canyet s/n, 08916 Badalona, Barcelona, Spain

**Keywords:** Biological medicine, Biosimilar, Disease-modifying antirheumatic drug, Drug utilization study, JAK inhibitor, Rheumatoid arthritis

## Abstract

**Objective:**

The objective of this study is to compare the effectiveness of reference biologic medicines used in the treatment of rheumatoid arthritis (RA) specifically adalimumab, etanercept, and infliximab, with corresponding biosimilar medicines, based on an exploratory analysis of clinical data obtained in patients treated with these medicines in five hospitals in the region of Catalonia, Spain.

**Methods:**

There is a consultation of the database of the Registry of Patients and Treatments of the Catalan Health Service: extraction of data from adult patients diagnosed with moderate and severe active RA and with active prescription of at least one biological drug (reference or biosimilar) or JAK inhibitor. To compare the effectiveness of each reference biologic with its biosimilar, differences in mean DAS28-ESR values before and after treatment were assessed for adalimumab and its biosimilar, etanercept and its biosimilar, and infliximab and its biosimilar.

**Results:**

The study consisted of 643 patients. The most dispensed medicines were anti-TNFs, with 303 patients on treatment. Thirty-six percent of all patients were using biosimilars. No statistically significant differences were observed in any of the three comparisons between the reference biologic medicine and its biosimilar. These findings suggest that biosimilars have comparable effectiveness to reference biologics in reducing DAS28-ESR; in addition, they can provide substantial savings to public health systems.

**Conclusions:**

A significant number of patients diagnosed with moderate to severe active RA were treated with biological medicines and receiving the available biosimilar treatments. Future research should be conducted to confirm comparable effectiveness found to their reference biologic medicines in this exploratory analysis.

**Table:** 

**Key Points** • *Biosimilar use: 36% of rheumatoid arthritis (RA) patients in Catalonia are treated with biosimilars, exceeding the 12% recommendation. This reflects growing acceptance of these alternatives.* • *Comparative effectiveness: Biosimilars of adalimumab, etanercept, and infliximab showed comparable therapeutic benefit to their reference biologics in reducing disease activity in active rheumatoid arthritis.* • *Real-world data: The study provides real-world data from five hospitals, making biosimilar medicines a viable choice for rheumatologists in routine rheumatoid arthritis management.*

## Introduction

Chronic inflammatory rheumatic diseases such as rheumatoid arthritis (RA) represent a significant challenge for healthcare systems due to their disabling nature, progressive course, and substantial economic burden. Over recent decades, biologic therapies have transformed the clinical management of these conditions by providing more effective control of inflammation and substantially improving patients’ quality of life. However, the high cost of these innovative biological medicines has restricted access [1–4].

In this context, biosimilars have emerged as a highly relevant therapeutic alternative. A biosimilar is a biologic medicine that exhibits high similarity to a reference biological product in terms of quality, biological activity, safety, efficacy, and immunogenicity, although minor differences may exist due to the inherent complexity of biotechnological manufacturing processes. The introduction of biosimilars into the market holds the potential to significantly reduce the cost of biological treatments, foster competition within the pharmaceutical sector, and enhance the sustainability of healthcare systems [5].

In rheumatology in particular, where many patients require long-term biologic therapy—often in combination with other immunomodulatory agents—the availability of biosimilars represents a critical opportunity to expand access without compromising the quality of care. Nevertheless, to ensure the confidence of healthcare professionals and patients, rigorous comparative studies are essential to demonstrate the equivalence of a biosimilar to its reference product. These studies must encompass clinical, pharmacodynamic, pharmacokinetic, and immunogenicity parameters, in accordance with international regulatory standards [6, 7].

RA is a multifactorial, systemic autoimmune disease of unknown etiology. It is characterized by chronic inflammation primarily affecting the synovial joints, leading to pannus formation, progressive bone erosion, and ultimately, joint destruction [1, 2]. RA tends to be more active during its early stages and, if left untreated or poorly managed, progresses to joint deformity and irreversible damage [3, 4]. The most prominent clinical symptom is pain, which significantly affects patients’ quality of life and may eventually necessitate joint replacement or orthopedic surgery [5]. Beyond the joints, the persistent systemic inflammation associated with RA may cause a wide range of extra-articular manifestations and comorbidities [1–4].

According to the EPISER 2016 study on the epidemiology of rheumatic diseases in Spain [8], the estimated prevalence of RA in the adult population is 0.82% (95% CI: 0.59–1.15). This figure is relatively high compared to other countries with similar demographic and healthcare characteristics. The prevalence is notably sex-specific, with a significantly higher rate observed in women (1.54%) compared to men (0.57%).

The principal aim of RA treatment is to suppress inflammation, alleviate symptoms such as pain, swelling, and stiffness, and prevent long-term joint damage and deformity. Equally important is the preservation of function and quality of life, reducing the risk of disability and increasing life expectancy. Treatment strategies also target the prevention of complications such as cardiovascular disease and osteoporosis, which are common in RA patients. A comprehensive, multidisciplinary approach is recommended, combining pharmacological interventions with lifestyle modifications, patient education, and physical therapy. Among pharmacologic treatments, early initiation of therapy with nonsteroidal anti-inflammatory drugs (NSAIDs), glucocorticoids, and disease-modifying antirheumatic drugs (DMARDs) is essential. Among DMARDs, methotrexate (MTX) remains the cornerstone therapy due to its well-established effectiveness and favorable risk–benefit profile [3, 9–11]. In cases where conventional DMARDs alone are insufficient, biologic agents are introduced—often in combination with MTX—to enhance clinical outcomes. This combination has shown increased effectiveness and durability of response, while also delaying structural joint damage, making it a preferred therapeutic strategy [9–11].

At the time of the study, nine biologic agents were available in Spain as first-line options for the treatment of moderate to severe RA [12]. These agents can be classified based on their mechanism of action: anti-TNF-α action, *infliximab*, *adalimumab*, and *golimumab*, which are monoclonal antibodies [13, 14]; *certolizumab pegol*, a PEGylated Fab′ fragment of a humanized antibody [15, 16]; etanercept, a fusion protein dimer [9, 17]; T-cell co-stimulation modulators: such as abatacept [18]; IL-6 receptor antagonists: including tocilizumab and sarilumab [19, 20]; and IL-1 inhibitors: such as anakinra, although its clinical use is limited due to lower comparative effectiveness [12, 21]. *Rituximab*, a B-cell depleting monoclonal antibody, is also approved for second-line use in patients who do not respond adequately or are intolerant to other biologics or DMARDs. Additionally, targeted synthetic DMARDs such as Janus kinase (JAK) inhibitors—including tofacitinib and baricitinib—offer an oral treatment alternative with proven effectiveness [22, 23].

The Spanish Society of Rheumatology (SER) has developed a set of thirteen consensus-based clinical recommendations for the use of DMARDs and biologics in adult RA patients [24]. Risk management protocols specific to biologic therapies have also been published by SER [25]. Internationally, organizations such as the American College of Rheumatology (ACR) have issued updated guidelines, such as the 2021 ACR recommendations, to guide clinical decision-making and promote standardized care [5].

The overarching therapeutic goal in RA is disease remission. This is assessed using validated composite indices, with the Disease Activity Score (DAS) endorsed by the European League Against Rheumatism (EULAR) as a standard tool. Specifically, the DAS28-ESR index incorporates clinical evaluations of 28 joints, patient-reported health assessments, and erythrocyte sedimentation rate (ESR) values to gauge disease activity and treatment response [11, 26, 27].

In the region of Catalonia, access to biologic therapies is regulated under the Pharmacotherapeutic Harmonization Program (PHF), which establishes specific criteria for their prescription in patients with moderate to severe active RA [28]. These criteria align with the guidelines set by the Spanish Society of Hospital Pharmacy (SEFH) [29] and are detailed in documents issued by relevant regional pharmacotherapeutic committees, such as the CFT-MHDA and the CFT-SISCAT [12, 19, 22, 30]. The PHF seeks to ensure the rational use of medications by optimizing therapeutic effectiveness, safety, and efficiency [31].

The introduction of biosimilar medicines has played a significant role in reducing healthcare costs associated with RA treatment. For instance, in 2020, the average cost of anti-TNF-α therapies in Spain dropped by approximately 17% following the introduction of biosimilars [32]. The SEFH has confirmed that biosimilars offer comparable quality, safety, and effectiveness to their originator biologics, but at a lower price point [6, 33]. This has facilitated broader access to advanced therapies and improved the sustainability of healthcare systems managing chronic inflammatory conditions.

The primary objective of this study was to compare the clinical effectiveness of reference biologic medicines—specifically infliximab, etanercept, and adalimumab—and their corresponding biosimilars in the treatment of active RA. This evaluation was conducted through an exploratory analysis of real-world clinical data collected from patients with RA treated at five hospitals in Catalonia (Spain).

## Methods

The Strengthening the Reporting of Observational Studies in Epidemiology (STROBE) statement (https://www.strobe-statement.org/checklists/) was followed in this study.

### Study design and scope

A descriptive cross-sectional multicenter study was carried out in five hospitals in the province of Barcelona (Spain).

### Population and sample

Patients are diagnosed with active RA and with an active prescription for at least one biological medicine (reference or biosimilar) or JAK inhibitor, as of January 8th 2020 (the cut-off date chosen for the cross- sectional study).

Inclusion criteria were: adult patients with moderate to severe active RA based on the use of DAS28 (moderate activity, severe activity) [27], with an inadequate response, such as ineffectiveness or intolerance, to conventional DMARDs (including MTX), according to the SER [23, 24], who were on active treatment at the date of data extraction and with a last recorded date of visit to the Rheumatology Service after October 8th 2019.

The exclusion criteria were as follows: patients under 18 years of age, oncology patients, patients who did not attend the follow-up appointment at the Rheumatology Service at least 3 months before the cross-sectional cut-off date, and patients on biological therapy treatment in services other than rheumatology.

### Source of data collection and study variables

The information was obtained from the Register of Patients and Treatments (RPT) of the Catalan Health Service (CatSalut), in the category of Medicines for Outpatient Hospital Medication (MHDA), which is available via the applications portal of the Generalitat de Catalunya Health Department.

The studied variables were as follows: hospital (health center where patients were treated), demographic variables (age and sex), synthetic and/or biological medicines taken by the patient before the date of data extraction, current biological treatment (reference or biosimilar to analyze biosimilar medicines and reference biological medicines separately), and biosimilar prescribing physicians (in total and in each hospital to perform a comparative analysis to characterize the trend in the prescription of biosimilar medicines together with the role of the medical professional in promoting the use of these medicines). The targeted biological and synthetic medicines that these patients were receiving, i.e., the treatment of choice, were analyzed and classified according to the Anatomical, Therapeutic, Chemical Classification System (ATC). Another studied variable was the DAS28-ESR value before starting biological treatment and after biological treatment (value at the last medical visit prior to data extraction). The DAS28-ESR was interpreted as follows: less than 2.6, disease remission; from 2.6 to < 3.2, low disease activity; and between 3.2 and 5.1, moderate disease activity; greater than 5.1, high disease activity [24, 26, 27]. PHF recommends that the patient be on the medication for 12 months before assessing response to determine whether there are differences between reference biological medicines, biosimilars, or JAK inhibitors [28].

### Statistical analysis

A descriptive analysis was performed, expressing discrete variables as proportions and continuous variables as mean ± standard deviation. Normality was assessed using the Shapiro–Wilk test for variables with fewer than 30 cases and the Kolmogorov–Smirnov test for the remaining variables. Bivariate analysis included the Chi-square test for frequencies and Student’s *t*-test or ANOVA for means, with the Wilcoxon test as a non-parametric alternative. Fisher’s exact test was used to analyze DAS28-ESR status evolution. In cases where statistically significant differences were not verified due to small sample sizes, more robust methods such as bootstrapping were employed. Statistical significance was set at *p* < 0.05, and analyses were conducted using R software (v4.2.2).

### Ethical aspects

According to Spanish regulation (Order SAS/3470/2009, of December 16, which publishes the guidelines on post-authorization studies of an observational type for medicines for human use and Chapter VI of Royal Decree 577/2013, of July 26, which regulates pharmacovigilance of medicinal products for human use), studies prior to January 2021 were not legally required to obtain permission from the Ethical Review Committee or register the study protocol. Instead, a protocol was developed and approved in March 2020 (code number 20204002) by the Research Committee of one of the hospitals of this multicenter study (Hospital General de Granollers, Granollers, Barcelona, Spain).

The extraction of information was carried out anonymously, and the relationship was not available to recover which real cases the information corresponds to. Informed consent was waived since an Ethical Review Committee approval of a protocol was not required at the time of the study.

## Results

### Demographic variables

The study was conducted in 838 patients with active RA and being treated with at least one biologic (reference or biosimilar) medicine or JAK inhibitor, after demonstrating a lack of response or intolerance to MTX or another previously prescribed conventional DMARD. One hundred ninety-five patients were excluded because they did not meet the inclusion criteria and/or did not attend the follow-up consultation in the Rheumatology Service at least 3 months before the cross-section.

**Fig. 1:**
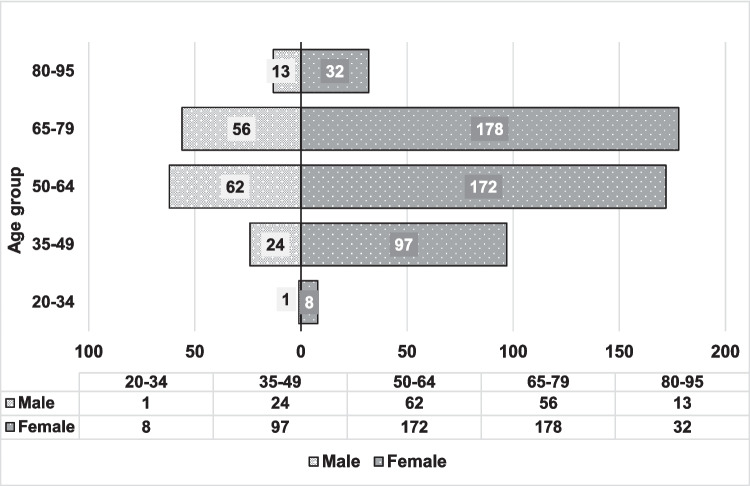
Distribution in the form of a population pyramid, according to sex and age, of the patients included in the study

Finally, 643 adult patients over 18 years of age with active biological treatment were included. Of the total number of patients included in the study, 487 were women (75.8%) and 156 men (24.3%); in addition, 60.3% were under 65 years of age (minimum value 20), and the remaining 39.7% were over 65 years of age (maximum value 92) (Fig. 1). The mean age was 61.3 ± 13.1 years.

### Treatment analysis

All drugs belonged to the ATC group of antineoplastic and immunomodulators and, within this, to immunosuppressive agents (L04). The most commonly used were tumor necrosis factor-alpha (TNF-α) inhibitors, followed by JAK inhibitors and interleukin inhibitors (Table 1).

**Table 1:** Pharmacotherapeutic treatments prescribed to patients according to Anatomical Therapeutic Chemical (ATC) classification system

Pharmacological group	ATC	Patients (*n*)
TNF-α inhibitors	**L04AB**	**303**
Adalimumab	L04AB04	90
Certolizumab pegol	L04AB05	47
Etanercept	L04AB01	118
Golimumab	L04AB06	31
Infliximab	L04AB02	17
JAK inhibitors	**L04AF**	**132**
Baricitinib	L04AF02	74
Tofacitinib	L04AF01	58
Interleukin inhibitors	**L04AC**	**119**
Anakinra	L04AC03	2
Sarilumab	L04AC14	35
Tocilizumab	L04AC07	82
Selective immunosuppressants	**L04AA**	**89**
Abatacept	L04AA24	89

**Fig. 2:**
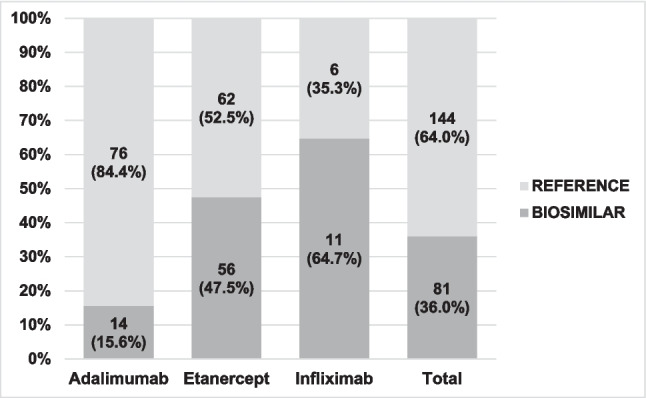
Use of biosimilar anti-TNF versus reference anti-TNF

In this study, 144 patients were being treated with adalimumab, etanercept, and infliximab (only these three biological medicines had biosimilars marketed at the time of the study). Only 81 patients were being treated with their respective biosimilars (36% of the total 225). When broken down by medicine, the percentage of biosimilars represented 15.5%, 47.4%, and 64.7% for adalimumab, etanercept, and infliximab, respectively (Fig. 2).

**Fig. 3:**
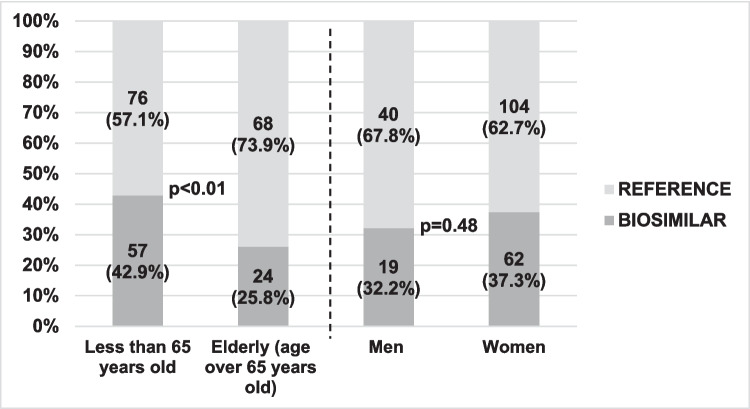
Percentage of biosimilar medicines used by age and sex

Table 2 shows the list of the 643 patients used for the study, indicating the drug, sex, and age range distribution. Significantly more frequent use was observed in patients younger than 65 years (42.9%), compared to 26.1% in those over 65 years of age (*p* < 0.01). Higher use in women (37.4%) than in men (32.3%), although without statistical significance (*p* = 0.48) (Fig. 3).

**Table 2:** Number of patients receiving each medicine, distributed by sex and age range

Medicine	Patients (*n*)	Man (*n*)	Woman (*n*)	Age (*n*)				
				20–34 years	35–49 years	50–64 years	65–79 years	80–95 years
**Adalimumab**	76	20	56	2	9	32	27	6
***Adalimumab biosimilar***	*14*	*2*	*12*	*1*	*4*	*6*	*3*	*0*
**Certolizumab pegol**	47	10	37	4	20	9	10	4
**Etanercept**	62	17	45	0	10	21	26	5
***Etanercept biosimilar***	*56*	*15*	*41*	*0*	*2*	*5*	*7*	*1*
**Golimumab**	31	9	22	0	5	11	11	4
**Infliximab**	6	3	3	0	0	2	4	0
***Infliximab biosimilar***	*11*	*2*	*9*	*0*	*2*	*6*	*3*	*0*
**Baricitinib**	74	15	59	0	15	28	27	4
**Tofacitinib**	58	12	46	0	6	25	20	7
**Anakinra**	2	0	2	0	0	1	1	0
**Sarilumab**	35	7	28	2	13	9	10	1
**Tocilizumab**	82	17	65	0	9	33	37	3
**Abatacept**	89	25	64	0	12	29	38	10
**Total**	643	154	489	9	107	217	224	45

Thus, 25 of the 30 rheumatologists analyzed had prescribed one of the three alternative biosimilars registered in Spain (*adalimumab*, *etanercept* and *infliximab*); 17 of the 30 (56.7%) had prescribed a biosimilar medicine at least once, and the remaining 13 specialists (43.4%) had not prescribed any.

### Analysis of disease evolution

**Fig. 4:**
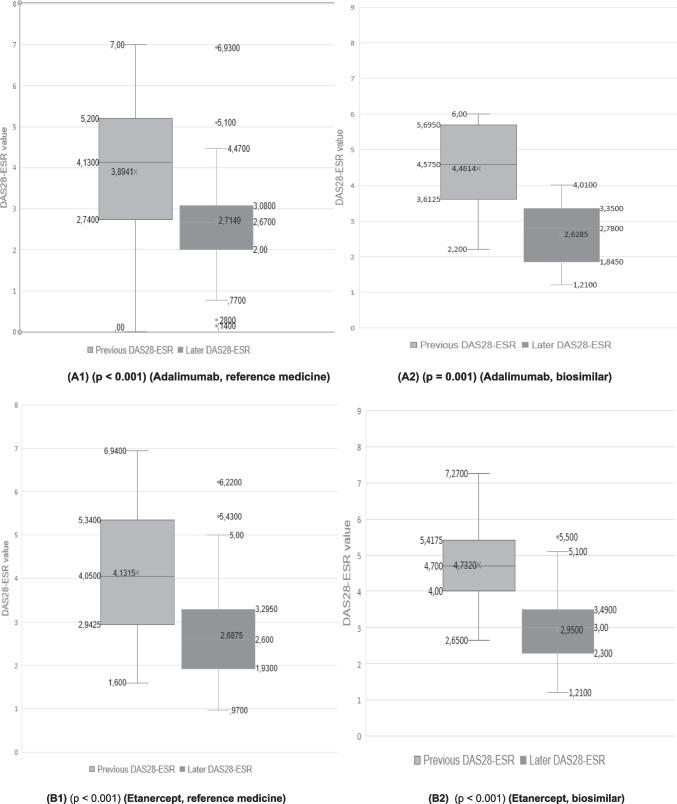

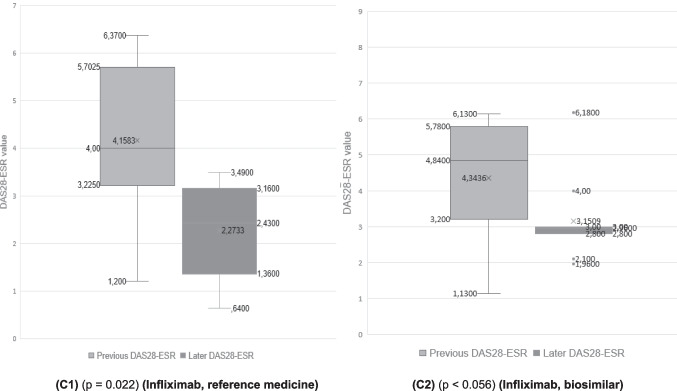
Quantitative analysis (box plots: **A**-**C**) of pre- (first value in the medical record) and later- (last value available in the medical record) DAS28-ESR values of different patient groups (**A1**) patients treated with the reference medicine adalimumab, (**A2**) patients treated with the adalimumab biosimilar, (**B1**) patients treated with the reference medicine etanercept, (**B2**) patients treated with the etanercept biosimilar, (**C1**) patients treated with the reference medicine infliximab, and (**C2**) patients treated with the infliximab biosimilar

In the case of patients who were treated with adalimumab (reference biological medicine) the mean value of the pre-treatment DAS28-ESR was 3.89. After treatment, the value dropped to 2.71 (post-treatment DAS28-ESR value). In the case of its biosimilar, the improvement of the disease even presented a better evolution, since the pre-treatment value DAS28-ESR was 4.46 and became 2.63. Statistically significant differences were found between the pre- and post-treatment DAS28-ESR values (Fig. 4(A1) and (A2)).

The comparison of etanercept (reference biological medicine) with its biosimilar indicated something similar. The mean DAS28-ESR value of the patients before treatment was 4.13, and once treated, it became 2.69 (a decrease of 1.44 points). In the case of patients who were treated with the etanercept biosimilar, the DAS28-ESR value went from 4.73 to 2.95 (a decrease of 1.78). It should be noted that the drop in the DA28-ESR value was greater in the case of biosimilars than for the reference medicines, which indicates their adequate effectiveness. Statistically significant differences were found between the pre- and post-treatment DAS28-ESR values (Fig. 4(B1) and (B2))*.*

Finally, we compared infliximab (reference biological medicine) with its biosimilar. The mean pre-treatment value DAS28-ESR was 4.15 and low to 2.27 post-treatment, once patients were treated with infliximab. In the case of its biosimilar, the mean value of DAS28-ESR went from 4.34 to 3.15, which represented a decrease of 1.88 points. In the case of the infliximab biosimilar, no statistical significance was reached (*p* = 0.056), although the *p*-value is very close to the conventional threshold of 0.05. This may be due to the low sample size, which limits the power of the test and increases the risk of type II error. In addition, the Wilcoxon test, being based on ranges and not absolute magnitudes, is less sensitive in small samples.

To explore this possible limitation, a non-parametric bootstrap analysis was applied to the pre-post difference of this group. The 95% confidence interval for the mean of the differences was [0.19, 2.19], which does not include null (0). This result indicates that, although the Wilcoxon test did not reach conventional significance, there is statistical evidence of significant improvement after treatment with the biosimilar infliximab (Fig. 4(C1) and (C2)).

However, the comparison of adalimumab and its biosimilar, etanercept and its biosimilar, and infliximab and its biosimilar, showed no statistically significant differences in any of the three comparisons between the reference biologic and its biosimilar (probability values of 0.279, 0.267, and 0.401, respectively). These findings suggest that biosimilars had an effectiveness similar to that of reference biologics.

**Fig. 5:**
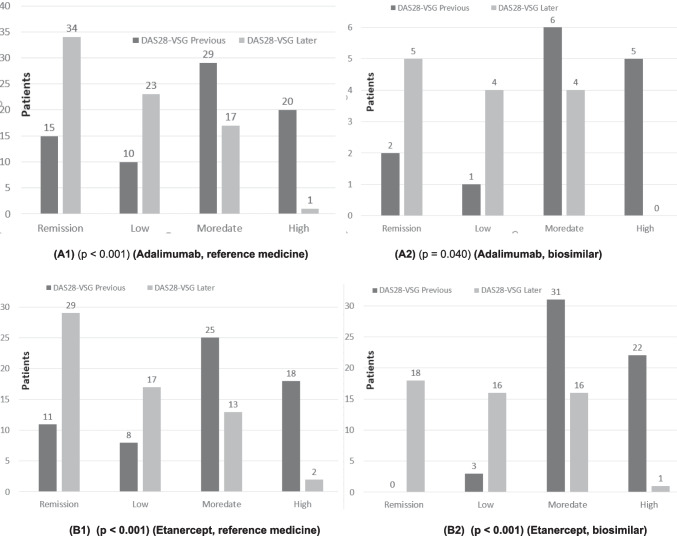

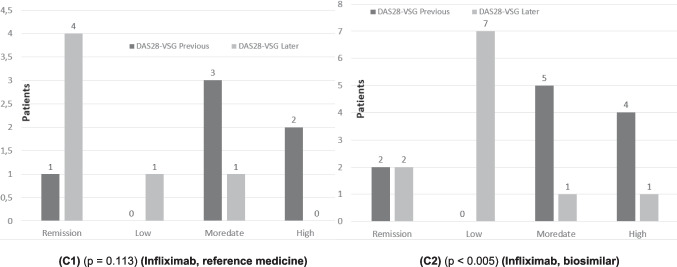
Qualitative analysis (box plots: **A**-**C**) of previous (first value in the medical record) and later (last value available in the medical record) DAS28-ESR values of different patient groups (**A1**) patients treated with the reference medicine adalimumab, (**A2**) patients treated with the adalimumab biosimilar, (**B1**) patients treated with the reference medicine etanercept, (**B2**) patients treated with the etanercept biosimilar, (**C1**) patients treated with the reference medicine infliximab, and (**C2**) patients treated with the infliximab biosimilar

With respect to the qualitative aspects, changes in the clinical status of the disease were analyzed according to the different treatments, using an ordinal classification (0 = remission, 1 = low activity, 2 = moderate activity, and 3 = high activity). The results showed statistically significant differences in the evolution of disease status for most treatments, except for infliximab (*p* = 0.113). The problem detected here is that the sample size was small in relation to the rest of the cases (only 6 patients). With a larger number of patients, possibly statistically significant differences would have been found. In the case of infliximab, bootstrapping was performed indicating that the pre-post-treatment differences were − 1.5. This result indicated a statistically significant improvement in disease status following treatment with infliximab, despite the small sample size (Fig. 5(A1) and (A2)).

However, the comparison of adalimumab and its biosimilar, etanercept and its biosimilar, and infliximab and its biosimilar showed no statistically significant differences in any of the three comparisons between the reference biologic and its biosimilar in relation to change in disease status (probability values of 0.901, 0.164, and 0.443, respectively). These findings suggest that biosimilars had similar effectiveness to reference biologics.

The analysis using the Fisher exact test allowed us to detect differences in the clinical course according to treatment. The absence of differences between reference medicines and biosimilars reinforces similar therapeutic benefit between them. The usefulness of the categorical approach to clinical status (remission, low, moderate or high activity) as a clinically relevant response variable was also confirmed.

## Discussion

The results presented in Fig. 2 show a lower use of biosimilars than reference medicines (36% versus 64%, respectively), a percentage that nevertheless exceeds the recommendation established in the Catalonian PHF [22], which was 12%, according to sources consulted at the Pharmacy Services in the hospitals included in the study [34].

The low utilization of biosimilars may be due to a lack of trust on the part of the patient and the physician. Frantzen et al. [35] found precisely this situation: after providing information about biosimilar medicines to 629 patients, only 25% reported feeling safe to be treated with these medicines. A study by Kolbe et al. [36], conducted in the USA, found that there were gaps in knowledge and hesitation among physicians in the US healthcare system when it came to prescribing biosimilars.

Of the three registered biosimilar medicines available in Spain at the time of the study, *etanercept* was the most prescribed medicine, accounting for 47% of biosimilar prescriptions. *Adalimumab*, with 12 biosimilar presentations, only had 16% of the biosimilar prescriptions. *Infliximab*, despite being the medicine that has been on the market for the longest time (1999), was the least dispensed medicine of these three.

The lower percentage of *adalimumab* prescriptions may be related to a greater distrust of this medicine, although several studies have supported the efficacy of its biosimilars [37]. The possible differences between the different *presentations of adalimumab* could be due to limitations in the studies and biases of the centers in which they were performed and highlight the similarity in both efficacy and safety of biosimilars with respect to the biological medicine. Other studies, such as that of Bruni et al. [38], also demonstrated the safety of the biosimilar *adalimumab* in joint and autoimmune diseases.

Among rheumatology specialists, 56.7% had prescribed a biosimilar medicine. This result is much higher than that found in the study by Delgado et al. [39], carried out in Europe during the 12 years to 2019, in which it was found that in Spain only 25% of doctors had ever prescribed a biosimilar medicine.

Currently, the European Medicines Agency (EMA) and the Heads of the Medicines Agencies have confirmed that biosimilar medicines have proven to be comparable to their reference products in terms of efficacy, safety, and immunogenicity and are therefore interchangeable [40].

Figures 4 and 5 show that patients who initiated treatment with *the reference adalimumab*, *biosimilar adalimumab*, *reference etanercept*, biosimilar etanercept, reference infliximab, and infliximab biosimilar in all cases improved the DAS28-ESR value. In some cases, this factor had been reduced, improving the patient’s life, and in other cases, the disease had disappeared.

This could indicate that these three biosimilar medicines are comparable to their respective reference biologic medicines in their effectiveness. Unfortunately, there are few similar studies—with the analysis of the DAS28 or DAS28-ESR factor as evidence of the evolution of the rheumatic disease—with which to compare and evaluate the results of this study. A retrospective study of a patient cohort from two local health boards in Wales was conducted to analyze the clinical outcomes in terms of DAS28 of *the etanercept biosimilar* compared to the reference of etanercept in real-world practice [41]. In this study, although the authors assume a reduction in DAS28 as an improvement in treatment in both groups, they criticized the lack of more specific measures of disease activity. A single-center retrospective observational study conducted in the UK by Madenidou et al. [42] also used DAS28 as a measure of treatment loss of effect, but in this case DAS28 was taken as a subjective measure.

This study does not include the ACR response criteria. The ACR score is the most commonly used outcome in clinical trials and allows for a common standard among researchers. In a systematic review, Konzett et al. [43] evaluated which ACR response definition (ACR20, 50, or 70) should be used primarily for efficacy claims in future RA medicine approval trials. But at the same time, their results support the selection of stricter thresholds if subsequent time points are to be evaluated, given their comparable but higher clinical validity. In Catalonia in real practice: the rheumatologist uses the DAS28 to measure the activity of the disease and the response to medicines. In fact, the CFT-MHDA of CFT-SISCAT uses DAS score to evaluate the levels of efficiency, effectiveness, and therapeutic utility of these medicines in their payment criteria [31].

This study wanted to explore if biosimilars were effective and safe medicines as reference products, being a viable choice for rheumatologists, with the aim of promoting their use in hospitals across Catalonia. This strategy was primarily motivated by economic considerations and the potential to generate substantial savings for the public healthcare system [44]. Multiple studies have shown that the use of biosimilars results in significant cost savings within public health systems. In a study similar to ours, the adoption of biosimilars for adalimumab, infliximab, and etanercept in 44.6% of 178 treated patients led to a total saving of €213,530 [45].

Extrapolating this approach to the entire autonomous community of Catalonia—where the prevalence of RA and other immune-mediated diseases requiring biological therapy is estimated at 0.5–0.7% of the adult population (approximately 30,000 to 40,000 patients)—the potential annual savings could exceed €100 million, assuming similar biosimilar uptake rates. In an ideal scenario where all eligible patients were treated with biosimilars, the total economic benefit could range between €131 and €183 million per year, based on an average saving of €3,500 per patient. These projections underscore the considerable economic and strategic value of expanding biosimilar adoption as a means to enhance the sustainability and efficiency of the public healthcare system, without compromising treatment effectiveness or patient safety. This highlights the significant potential of biosimilars to contribute to a more efficient and resilient healthcare model.

As a general assessment, the results we present correspond to a descriptive, cross-sectional, multicenter study. Cross-sectional designs, in particular, have limitations: they provide only a snapshot in time, limiting the assessment of changes or trends; they cannot establish causal relationships; and they are susceptible to various biases (e.g., selection or information bias). However, this study reflects what happens in routine clinical practice, being useful for identifying associations and generating hypotheses. Besides, the multicenter setting also enhances the generalizability.

## Conclusions

This study conducted in five hospitals in Catalonia provides an updated perspective on the use of biological therapies in patients with moderate to severe RA. The findings revealed that a significant number of patients are being treated with biological medicines, including biosimilars. Despite some initial mistrust toward the latter, clinical experience seems to indicate that patients obtain benefits of biosimilar medicines.

The analysis of real-world data suggests a similar effectiveness between reference biological medicines and their biosimilar counterparts, with evidence of clinical improvement and symptom reduction. These preliminary results support the potential of biosimilars as effective and cost-efficient therapeutic alternatives.

Further research is warranted to confirm these findings. Such studies will allow for the evaluation of the therapeutic performance and economic impact of biosimilars under routine clinical conditions and in broader, more representative patient populations.

## Data Availability

The datasets used and/or analyzed during the current study are available from the corresponding authors on reasonable request.

## Abbreviations

*ACR*: American College of Rheumatology
*ANOVA*: Analysis of variance test
*ATC*: Anatomical Therapeutic Chemical Classification
*CatSalut*: Catalan Health Service
*CFT-MHDA*: Pharmacotherapeutics Committee for Outpatient Hospital Medication
*CFT-SISCAT*: Pharmacotherapeutics Commission - Integrated Public Healthcare System of Catalonia
*DAS*: Disease Activity Score
*DAS28-ESR*: Disease Activity Score 28-Erythrocyte Sedimentation Rate
*DMARDs*: Disease-modifying antirheumatic drugs
*EMA*: European Medicines Agency
*ESR*: Erythrocyte sedimentation rate
*EULAR*: European League Against Rheumatism
*IL-1*: Interleukin-1
*IL-6*: Interleukin-6
*JAK*: Janus kinase family protein signaling pathway
*MHDA*: Outpatient Hospital Medication
*MTX*: Methotrexate
*NSAIDs*: Non-steroidal anti-inflammatory drugs
*PHF*: Pharmacotherapeutic Harmonization Program
*RA*: Rheumatoid arthritis
*RPT*: Register of Patients and Treatments
*SEFH*: Spanish Society of Hospital Pharmacy
*SER*: Spanish Society of Rheumatology
*TNF-α*: Tumor necrosis factor-alpha
